# Tremorgenic effects and functional metabolomics analysis of lolitrem B and its biosynthetic intermediates

**DOI:** 10.1038/s41598-019-45170-7

**Published:** 2019-06-27

**Authors:** Priyanka Reddy, Simone Rochfort, Elizabeth Read, Myrna Deseo, Emily Jaehne, Maarten Van Den Buuse, Kathryn Guthridge, Martin Combs, German Spangenberg, Jane Quinn

**Affiliations:** 1 Agriculture Victoria, AgriBio, Centre for AgriBioscience, Bundoora, Victoria 3083 Australia; 2 School of Applied Systems Biology, La Trobe University, Bundoora, Victoria 3083 Australia; 3 School of Psychology and Public Health, La Trobe University, Bundoora, Victoria 3083 Australia; 4 School of Animal and Veterinary Sciences, Charles Sturt University, Wagga Wagga, NSW 2678 Australia; 5 Graham Centre for Agricultural Innovation, Charles Sturt University, Wagga Wagga, NSW 2650 Australia

**Keywords:** Neurodegeneration, Metabolomics

## Abstract

The neuroactive mycotoxin lolitrem B causes a neurological syndrome in grazing livestock resulting in hyperexcitability, muscle tremors, ataxia and, in severe cases, clonic seizures and death. To define the effects of the major toxin lolitrem B in the brain, a functional metabolomic study was undertaken in which motor coordination and tremor were quantified and metabolomic profiling undertaken to determine relative abundance of both toxin and key neurotransmitters in various brain regions in male mice. Marked differences were observed in the duration of tremor and coordination between lolitrem B pathway members, with some showing protracted effects and others none at all. Lolitrem B was identified in liver, kidney, cerebral cortex and thalamus but not in brainstem or cerebellum which were hypothesised previously to be the primary site of action. Metabolomic profiling showed significant variation in specific neurotransmitter and amino acid profiles over time. This study demonstrates accumulation of lolitrem B in the brain, with non-detectable levels of toxin in the brainstem and cerebellum, inducing alterations in metabolites such as tyrosine, suggesting a dynamic catecholaminergic response over time. Temporal characterisation of key pathways in the pathophysiological response of lolitrem B in the brain were also identified.

## Introduction

‘Perennial ryegrass staggers’ is associated with ingestion of perennial ryegrass (*Lolium perenne* L.) pastures containing the indole diterpenoid toxin lolitrem B^1^. The toxin is produced by an endophytic fungus, *Epichloë festucae* var. *lolii*, that lives symbiotically within its perennial ryegrass host. The syndrome has been well documented in livestock and other herbivores^2–4^ and isolated lolitrem B has been shown to cause a sustained tremor response in both sheep and mouse models of the disease^5–7^. Pastures associated with toxic outbreaks contain a number of potentially neuroactive metabolites of the lolitrem biosynthetic pathway^8^ and currently their role in the presentation, severity and duration of the clinical signs, their location, and mode of action in the brain has not been well defined.

**Figure 1:**
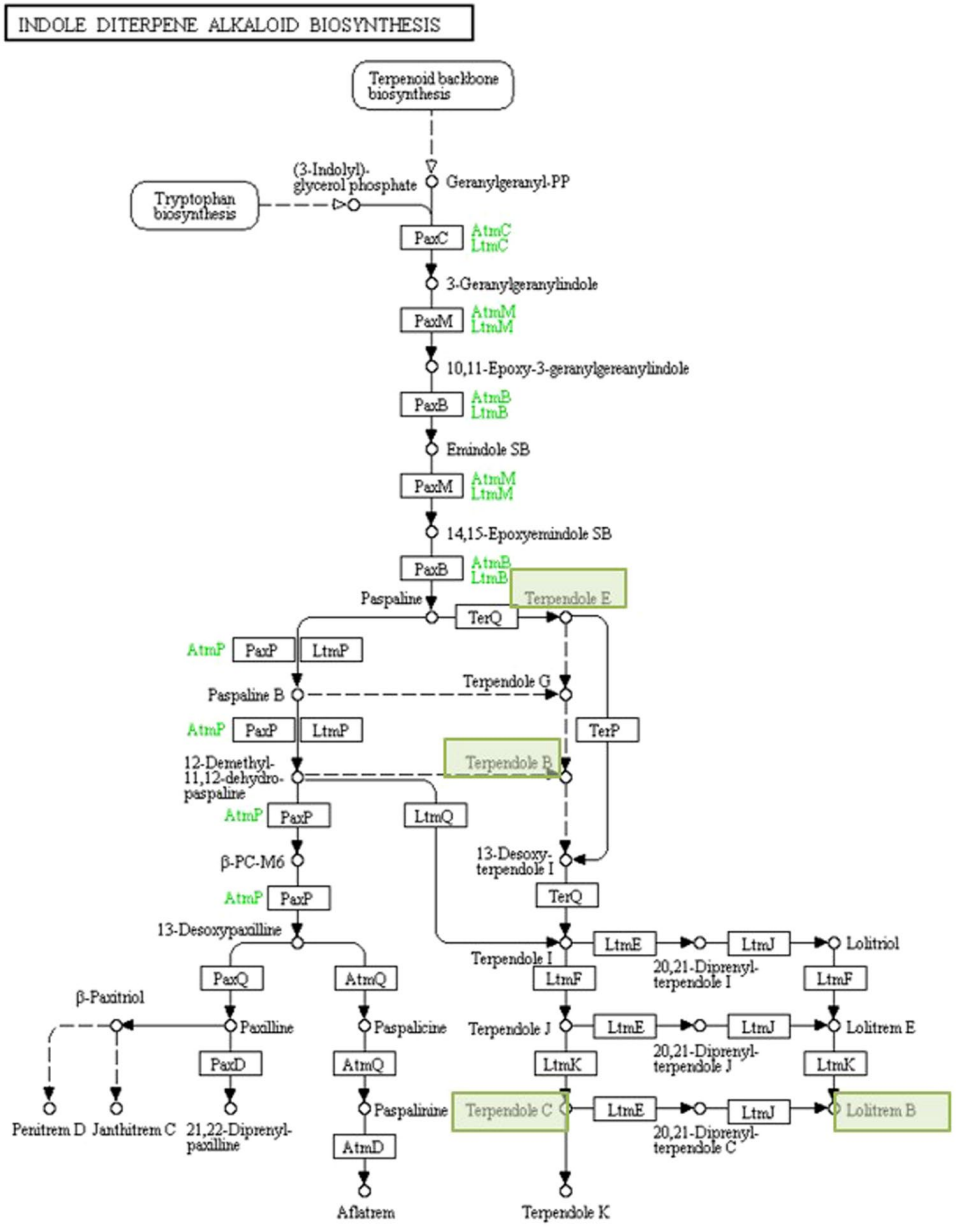
A biosynthetic map of lolitrem B sourced from KEGG pathways^68–70^ highlighting selected compounds tested for tremorgenic and motor deficit activity.

Lolitrem B is the final product in a complex biochemical pathway. The perennial ryegrass endophyte (*E*. *festucae* var. *lolii*) produces a number of important biochemical intermediates which also have biological functions, including protection from herbivory and drought tolerance^5,9–11^. The non-tremorgenic compounds paspaline and terpendole E^12^ represent a key divergence of two biosynthetic pathways, one of which will terminate with the tremorgenic diterpenoid toxins penitrem D^13^, janthitrem C^14,15^, aflatrem^5^ and the other which produces lolitrem B and terpendole K^16^ as endpoints (Fig. 1). Despite some of these compounds being well known as tremorgenic indole alkaloids, with lolitrem B identified as a long-acting and potent neurotoxin^5–7^, the activity of several pathway intermediates remains relatively unexplored.

The sustained tremors elicited by lolitrem B and its reversible response is considered to be a phenomena that has pharmacological significance^5^. The tremorgenic activity of lolitrems are suggested to be produced by the blockade of large conductance potassium channels (BK channels)^17^. BK channel inhibitors have many pharmaceutical applications, which have resulted in possible development of indole-diterpenes in drug design and discovery^17–21^. BK channels are found ubiquitously throughout the brain but are highly expressed in both the cerebellum and cerebral hemispheres^22,23^ and are suggested to constitute a primary target for these compounds. The cerebral cortex comprises a number of regions that regulate emotion, cognition, language, memory, homeostasis as well as auditory and visual areas^24^. BK channels are involved in the regulation of neurotransmitter release and neuronal excitability^23^ thus a blockade of these channels would cause downstream biological effects which have not yet been characterised in relation to lolitrems.

The inhibition of BK channel currents by the indole-diterpene molecules suggest that these receptors are most likely the major molecular target for these compounds. It has been reported that mice deficient in BK ion channels are unaffected by these neurotoxins at concentrations that are lethal to wild-type mice^17^. Also, the differences displayed by the *in vitro* interaction are in correlation with the *in vivo* response of these compounds, such as the duration of tremor exhibited and the affinity of the compounds to the receptor. This suggests that motor function deficits induced by lolitrems are mediated by BK channels^17^. However, lolitrem E and paspalicine although showing potent BK channel activity, elicit a non-tremorgenic effect on animals. This could be related to structural changes occurring *in vivo*, rendering it less active^25^. Knaus *et al*., suggested that although some pharmacological properties could be explained by BK channel inhibition, tremorgenicity may not be directly related to channel blockade^26^. Thus, it is possible that the relationship between tremor and BK channel activity is more of a complex interaction. This is further supported by the wide range of neurological observations in animals suffering from lolitrem B toxicosis, which are not restricted to movement, but include changes in behavior^4^. Thus, the hypothesis that lolitrem B affects, in part, neurotransmitter receptors leading to interference with neurotransmitter release in the central and peripheral nervous system needs to be examined.

Also, early work investigating the mode of action of the mycotoxins penitrem A and verruculogen shows that these toxins interfere with amino acid neurotransmitter release mechanisms in the central nervous system^27–29^. Together these data suggest region specific effects for this toxin in the brain, the effects of which have not been previously investigated.

To determine the effects of lolitrem B, and members of the indole diterpenoid biosynthesis pathway, on neurotransmitter and amino acid levels in the brain, a functional metabolomics study was undertaken. Adult mice were exposed to purified lolitrem B, and biosynthetic pathway members terpendole B, C and E, at two doses. Time to peak tremor was determined with tissue samples collected for analysis at the onset of peak tremor and 24 h later. Motor coordination was measured using the accelerating rotarod test^30,31^. Quantitation of concentration of lolitrem B in the brain was determined and neurotransmitter and amino acid expression compared in four key regions of the brain: cerebral cortex, thalamus, cerebellum and brainstem at the two time points. Principle components analysis (PCA) was undertaken to identify key determinants of effect.

This study showed that lolitrem B exerted region specific effects on neurotransmitter and amino acid levels in the brain of intoxicated mice with the cerebral cortex showing greatest changes in response to toxic exposure. These data suggest the cerebrum, a region that is involved in cognition, emotion and mental activity, to be an important site of action of lolitrem B toxin in affected animals. This hypothesis was further supported by the high levels of toxin found in this region. Furthermore, alteration in neurotransmitter levels observed in the lolitrem B-exposed brain *in vivo* suggest a primary mode of action of the toxin in catecholaminergic neurotransmitter pathway production, recycling or synthesis, a correlation that has not been previously demonstrated. This study identifies both region and metabolic specific action of the lolitrem B toxin in the brain, effects that may contribute to the clinical signs observed in animals affected by ingestion of toxic pastures or in controlled animal models.

## Results

**Figure 2:**
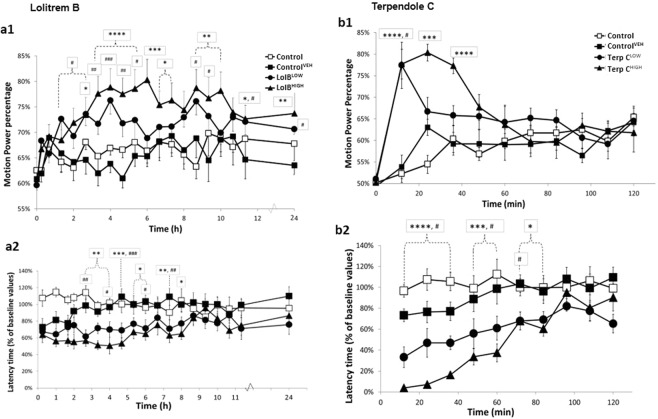
Lolitrem B intoxicated mice exhibit a dose-dependent tremor **(a1)** and latency to falling **(a2)** over 24 h time. Significant tremor and impaired motor coordination was measured for LolB^LOW^ (*n* = *8*) and LolB^HIGH^ (*n* *= 6*) compared to Control^VEH^ (*n* *= 7*). Terpendole C intoxicated mice monitored show a dose-dependent tremor **(b1)** and latency to falling **(b2)** over 2 h. Significant tremor and impaired motor coordination was measured for TerpC^LOW^ (*n* = *8*) and TerpC^HIGH^ (*n* = *8*) compared to Control^VEH^ (*n* = *7*). No significant tremor or motor impairment was found in the Control (*n* = *8*) animals. All data are mean ± S.E.M. *P* values determined by two-way ANOVA by uncorrected Fisher’s LSD post-test for multigroup comparison against vehicle control. High dose: *p < 0.05, **p < 0.01, ***p < 0.001, ****p < 0.0001; low dose: ^#^*p* < 0.05, ^##^*p* < 0.01, ^###^*p* < 0.001, ^####^*p* < 0.0001.

**Figure 3:**
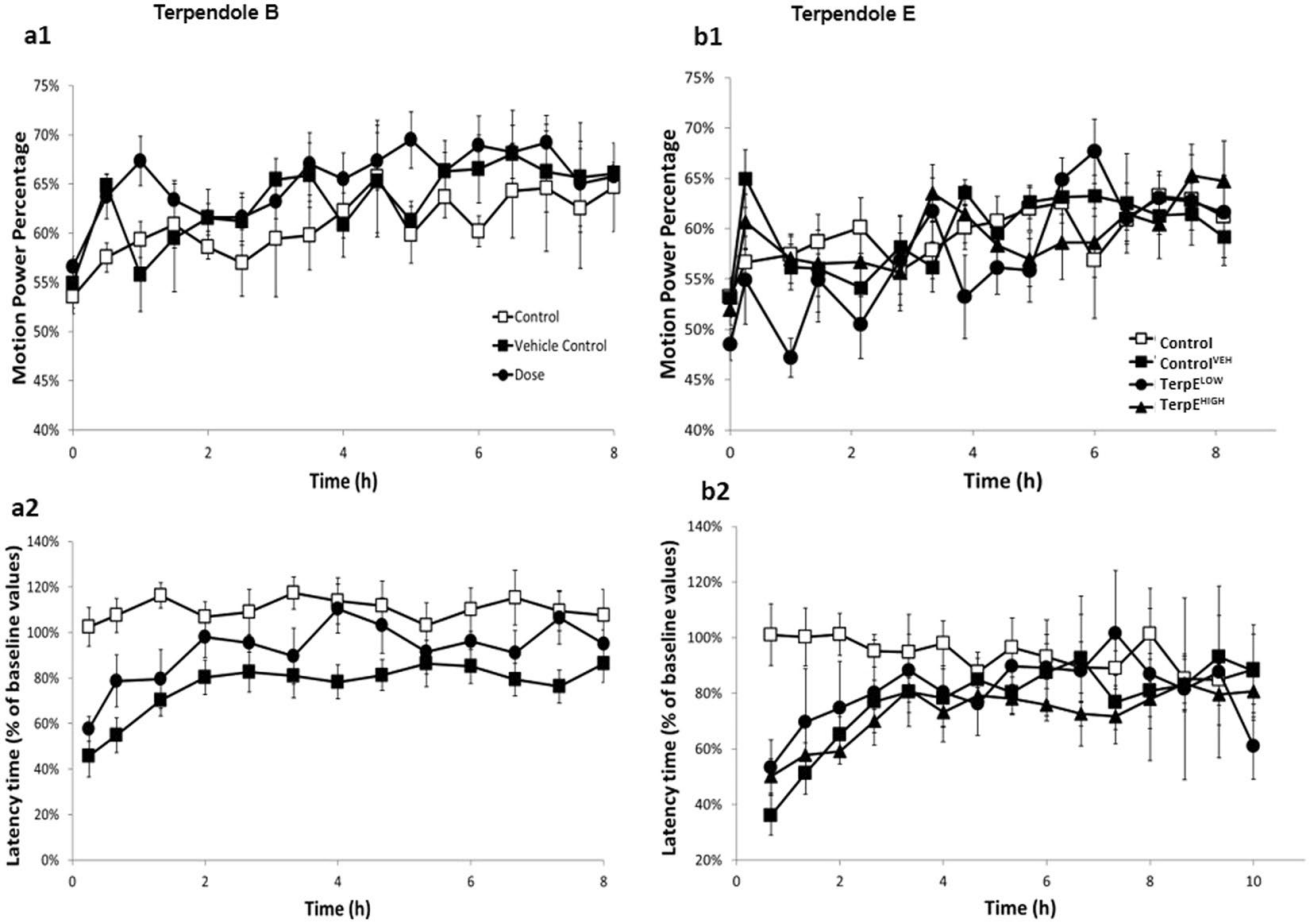
Terpendole B and terpendole E intoxicated mice exhibit no tremor **(a1**,**b1)** and latency to falling **(a2**,**b2)** over time. No significant tremor or alteration in motor coordination was observed between TerpE^LOW^ (*n* = *8*), TerpE^HIGH^ (triangle, *n* = *8*) and TerpB (*n* = *8*) compared to Control^VEH^ (*n* = *7*). No significant tremor or motor impairment was found in Control (*n* = *8*) mice. All data are mean ± S.E.M.

To determine the neurotoxic activity of lolitrem B pathway components, motor coordination and tremor were tested in response to treatment with four selected indole-diterpenoids, lolitrem B, terpendoles B, C and E (Figs 1–3).

Both LolB^HIGH^ and LolB^LOW^ mice showed increased motion power percentage (MPP) indicative of tremor (Fig. 2a1), compared to vehicle. Analysis by two-way ANOVA revealed a significant effect of treatment (*F*_*(3*,*560)*_ = 53.99; *p* < 0.0001) and of time (*F*_*(19*,*560)*_ = 4.15*; p* < 0.0001). However, the interaction between time and treatment was not significant (*F*_*(19*,*560)*_ = 1.125*; p* = 0.25) reflecting prolonged and sustained tremors detected over the 24 h period. Peak tremor occurred in LolB^HIGH^ mice at 6 h post treatment with animals exhibiting an MPP of 80% ± 0.04% compared to Control^VEH^ (MPP = 63% ± 0.02; *p* = 0.0003). LolB^HIGH^ mice showed tremors for the duration of the study and tremors remained significant at 24 h (MPP = 77 ± 0.04%) compared to Control^VEH^ (MPP = 64 ± 0.02%; *p* = 0.005). LolB^LOW^ mice showed peak tremor at 4 h post treatment (MPP of 76% ± 0.03%) compared to Control^VEH^ (64% ± 0.02; *p* = 0.0006). Tremors for LolB^LOW^ mice also lasted for 24 h post treatment (MPP = 71% ± 0.01) compared to Control^VEH^ (MPP = 65% ± 0.02; *p* = 0.049). A dose-dependent response was observed with LolB^HIGH^ mice showing significantly greater tremor response than LolB^LOW^ counterparts. Control and Control^VEH^ mice showed no tremor response at any time point tested.

Motor coordination was assessed in lolitrem B treated mice by accelerating rotarod testing (Fig. 2a2). Analysis by two-way ANOVA revealed a significant effect of treatment (*F*_*(3*,*440)*_ = 54.33; *p* < 0.0001) but no significant effect of time (*F*_*(19*,*440)*_ = 1.33*; p* = 0.16) or interaction between time and treatment (*F*_*(57*,*440*)_ = 1.16*; p* = 0.21). LolB^HIGH^ mice exhibited a significantly reduced latency to falling for the first eight time points of the testing period with peak effects observed at 2.7 to 5 h post treatment compared to Control^VEH^ (Time = 2.67 h, *p* = 0.005; 3.3 h, *p* = 0.007; 4 h, *p* = 0.002; 4.67 h, *p* = 0.0002 and 5.33 h, *p* = 0.030) (Fig. 2a2). LolB^LOW^ mice showed significant impairment of motor coordination for the first 6 h of the testing period with peak effects also observed from 2.7 to 4.7 h post treatment compared to Control^VEH^ (Time = 2.67 h, *p* = 0.002; 4 h, *p* = 0.031; 4.67 h, *p* = 0.0006) (Fig. 2a2). There was a significant difference between the two dose rates (Fig. 2a2). Control^VEH^ mice also showed impairment of motor coordination by rotarod testing up to 1 h 40 min post treatment compared to Control (Time = 0.25 h, *p* = 0.0091; 1 h, *p* = 0.012; 1.67 h, *p* = 0.037) suggesting that DMSO is not an inert carrier (Fig. 2a2).

Mice treated with terpendole C exhibited tremor but the profile of effect was markedly different to lolitrem B. Terpendole C has previously been identified to be a fast-acting tremorgen^32^. Analysis by two-way ANOVA revealed a significant effect of treatment (*F*_*(3*,*352*)_ = 7.444 *p* < 0.0001), time (*F*_(*10*,*352)*_ = 5.933*; p* < 0.0001*)* and interaction between time and treatment (*F*_*(30*,*352)*_ = 4.265*; p* < 0.0001*)*. Peak tremor occurred in TerpC^HIGH^ mice at 24 min post-treatment with mice exhibiting an MPP of 80% ± 0.06% compared to Control^VEH^ (63% ± 0.02; *p* < 0.0001). Significant tremor was recorded until 36 min post injection (Fig. 2b1) after which tremor rapidly reduced in power and had returned to baseline by 48 min. Peak tremor occurred in TerpC^LOW^ mice at 12 min post treatment with mice exhibiting an MPP of 77% ± 0.05% compared to Control^VEH^ (54% ± 0.03; *p* = 0.002). Although MPP% was similar in TerpC^LOW^ and TerpC^HIGH^ mice at 12 min post treatment, low dose mice rapidly returned to control levels by 24 min.

Similar to lolitrem B treated mice, loss of motor coordination was also observed in a dose-dependent manner for terpendole C treated mice (Fig. 2b2). Analysis by two-way ANOVA revealed a significant effect of treatment (*F*_*(3*,*308)*_ = 59.84; *p* < 0.0001), time (*F*_*(10*,*308)*_ = 11.13*; p* < 0.0001) and interaction between time and treatment (*F*_*(30*,*308)*_ = 2.657*; p* < 0.0001). TerpC^HIGH^ showed the greatest motor deficits from 12 to 36 min compared to Control^VEH^ (Time = 12 min, *p* < 0.0001, 24 min; *p* < 0.0001; 36 min, *p* < 0.0001) which is consistent with the tremor profile of this toxin. TerpC^LOW^ treated mice also exhibited significantly reduced latency to falling compared to Control^VEH^ with peak effects also observed in the first 36 min (Time = 12 min, *p* = 0.012; 24 min, *p* = 0.027 and 36 min, *p* = 0.042) (Fig. 2b2). However, in this study, observation of poor motor coordination persisted well after the period of measurable tremor with significant motor deficit recorded up to 84 min (*p* = 0.027) for TerpC^HIGH^ and 72 min (*p* = 0.019) for TerpC^LOW^ compared to Control^VEH^.

Two biosynthetic pathway intermediates in the lolitrem B biosynthesis pathway were tested for tremorgenic activity and motor coordination impairment. Terpendole E has previously been identified to be non-tremorgenic^33^ and is a biosynthetic precursor to terpendole B. Terpendole B has been previously isolated, however; its tremorgenic activity has not been previously investigated. Our data confirm that terpendole E is non-tremorgenic and reveal that terpendole B showed no significant tremorgenic activity at any dose tested (Fig. 3a1,b1). Also, no effects on motor coordination or function were observed for terpendole B and E (Fig. 3a2,b2).

**Figure 4:**
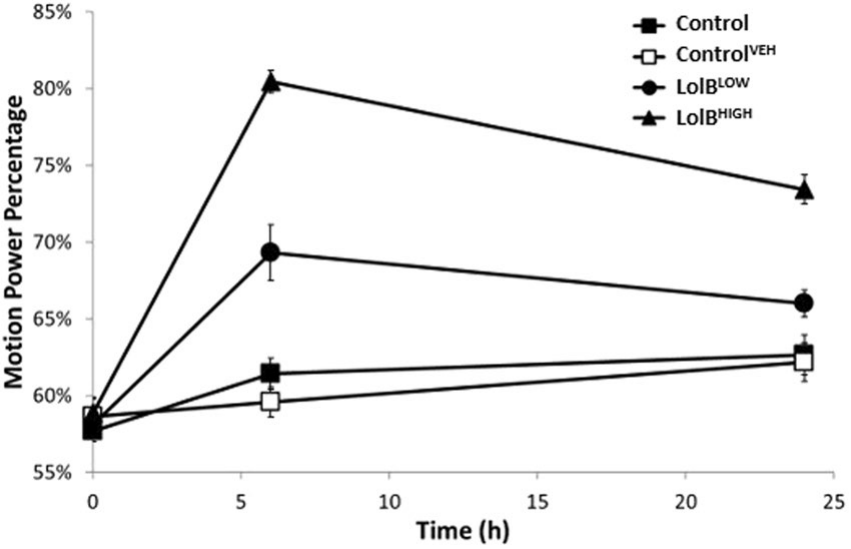
Lolitrem B intoxicated mice exhibit tremor at 6 h and 24 h post treatment. A dose-dependent response is observed for LolB^LOW^ (*n* = *8*) and LolB^HIGH^ (*n* = *7*). The profile here is similar to that observed previously (Fig. 2). No significant tremor was identified in the vehicle control (*n = 8*) and control (*n = 8*) mice. All data are mean ± S.E.M. Significance was analysed by two-way ANOVA by uncorrected Fisher’s LSD post-test for multigroup comparison against vehicle control, *p < 0.05, **p < 0.01, ***p < 0.001, ****p < 0.0001.

To confirm the tremor intensities ascertained for lolitrem B in the behavioural study (Fig. 2a1), tremor analysis was also carried out prior to sample collection for the metabolomics study across the two selected key time points (6 h and 24 h post injection) (Fig. 4). Analysis by two-way ANOVA revealed a significant effect of treatment (*F*_*(3*,*112)*_ = 32.65 *p* < 0.0001), time (*F*_*(3*,*112)*_ = 55.01*; p* < 0.0001) and interaction between time and treatment (*F*_*(9*,*112)*_ = 9.779*; p* < 0.0001). A similar tremor profile was observed in mice used for metabolomics profiling as compared to their counterparts in the behavioural study.

Although lolitrem B has been extensively postulated to exert its effect via interaction with BK channel receptors in the brain, presence of the parent compound or its bioactive metabolites, has not been confirmed in this tissue. To address this, quantitation of lolitrem B was undertaken in dissected brain tissue: cerebral cortex, thalamus, cerebellum and brain stem, and the distribution of toxin examined in comparison to levels found in the liver and kidney. Bioaccumulation, or elimination, were investigated over time in these tissues.

**Figure 5:**
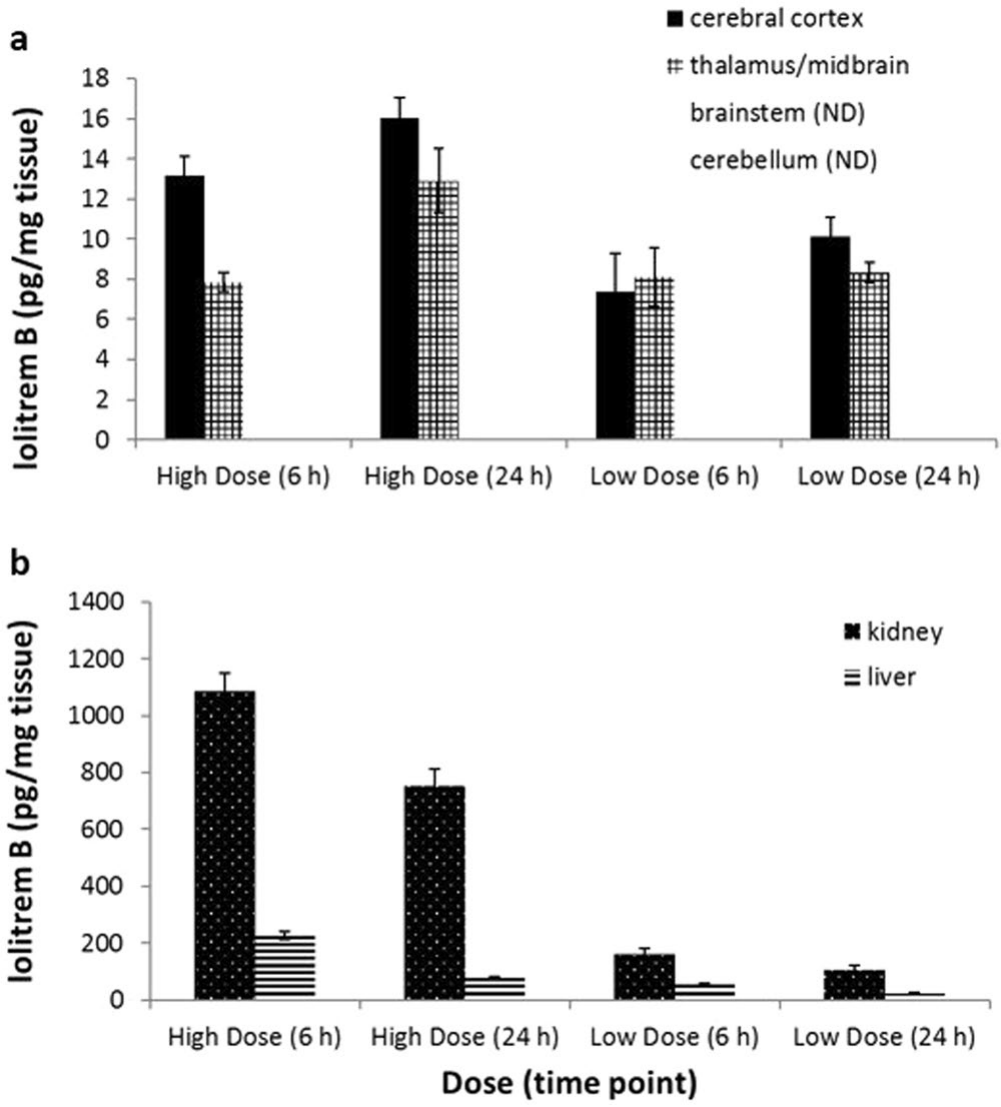
Lolitrem B concentrations in lipophilic extracts of tissues harvested at 6 h and 24 h post treatment **(a)** showing lolitrem B accumulation over time, in the cerebral cortex: LolB^HIGH^ [(6 h, *n* = *6*), (24 h, *n* = *8*)] and LolB^LOW^ [(6 h, *n* = *3*), (24 h, *n* = *4*)] as well as the thalamus: LolB^HIGH^ [(6 h, *n* = *7*), (24 h, *n* = *6*)] and LolB^LOW^ [(6 h, *n* = *3*), (24 h, *n* = *7*)]. Lolitrem B was below the detection limit in the cerebellum and brainstem regions. Graph **(b)** shows elimination of lolitrem B over time from the kidney: LolB^HIGH^ [(6 h, *n* = *7*), (24 h, *n* = *8*)] and LolB^LOW^ [(6 h, *n* = *7*), (24 h, *n* = *8*)], as well as from the liver: LolB^HIGH^ [(6 h, *n* = *7*), (24 h, *n* = *8*)] and LolB^LOW^ [(6 h, *n* = *8*), (24 h, *n = 8*)]. All data are mean ± S.E.M.

Quantitation by LCMS showed high levels of lolitrem B in the kidney and liver (Fig. 5b) with the highest concentrations observed in the liver 6 h post-treatement. Concentrations in the kidney (1084.4 ± 171.0 pg/g) were 5-fold higher than liver (201.5 ± 32.7 pg/g) with a clear dose-dependent effect and clear profile of excretion over time (Table 1). In LolB^HIGH^ mice at 6 h post treatment, lolitrem B was also identified in the cerebral cortex (13.2 ± 2.2 pg/g) and thalamus (8.8 ± 1.4 pg/g) (Table 1), at levels 58.6 and 13.9-fold lower than kidney and liver, respectively (Fig. 5a). No significant difference was observed in lolitrem B concentrations between cerebral cortex and thalamus, nor between high and low treatment groups or at any time points analysed. Together these data suggest an accumulation of lolitrem B specifically in cerebral cortex and thalamus brain regions. On acute exposure lolitrem B concentrates over 24 h in these brain regions; however, clearance may occur over a longer time period than analysed in this study. Lolitrem B was consistently below detectable limits in cerebellum and brainstem despite good recovery from these tissues (Extended Data is found in Supplementary Table S1).

**Table 1:** The mean concentration of lolitrem B (pg/mg of tissue) in body and brain tissues of mice exposed to LolB^HIGH^ and LolB^LOW^ at 6 h and 24 h post treatment.

Group	Kidney	Liver	Cerebral cortex	Thalamus	Brainstem	Cerebellum
LolB^HIGH^ (6 h)	1084.4 ± 171.0 *(n* = *7)*	201.5 ± 32.7 *(n* = *7)*	13.2 ± 2.2 *(n* = *7)*	8.8 ± 1.4 *(n* = *6)*	*ND*	*ND*
LolB^HIGH^ (24 h)	752.0 ± 171.8 *(n* = *8)*	76.7 ± 11.2 *(n* = *8)*	16.0 ± 2.8 *(n* = *8)*	12.9 ± 3.4 *(n* = *6)*	*ND*	*ND*
LolB^LOW^ (6 h)	162.0 ± 50.7 *n* = *8)*	53.6 ± 10.9 *(n* = *8)*	7.4 ± 3.3 *(n* = 3)*	8.1 ± 2.5 *(n* = 3)*	*ND*	*ND*
LolB^LOW^ (24 h)	107.2 ± 41.4 *(n* = *8)*	23.1 ± 7.5 *(n* = 5)*	10.1 ± 1.9 *(n* = 4)*	8.3 ± 1.4 *(n* = 7)*	*ND*	*ND*

*Some replicates in the LolB^LOW^ cohorts were found to contain below LoD (0.8 ng/mL levels of lolitrem B. ND-Not detected.

**Figure 6:**
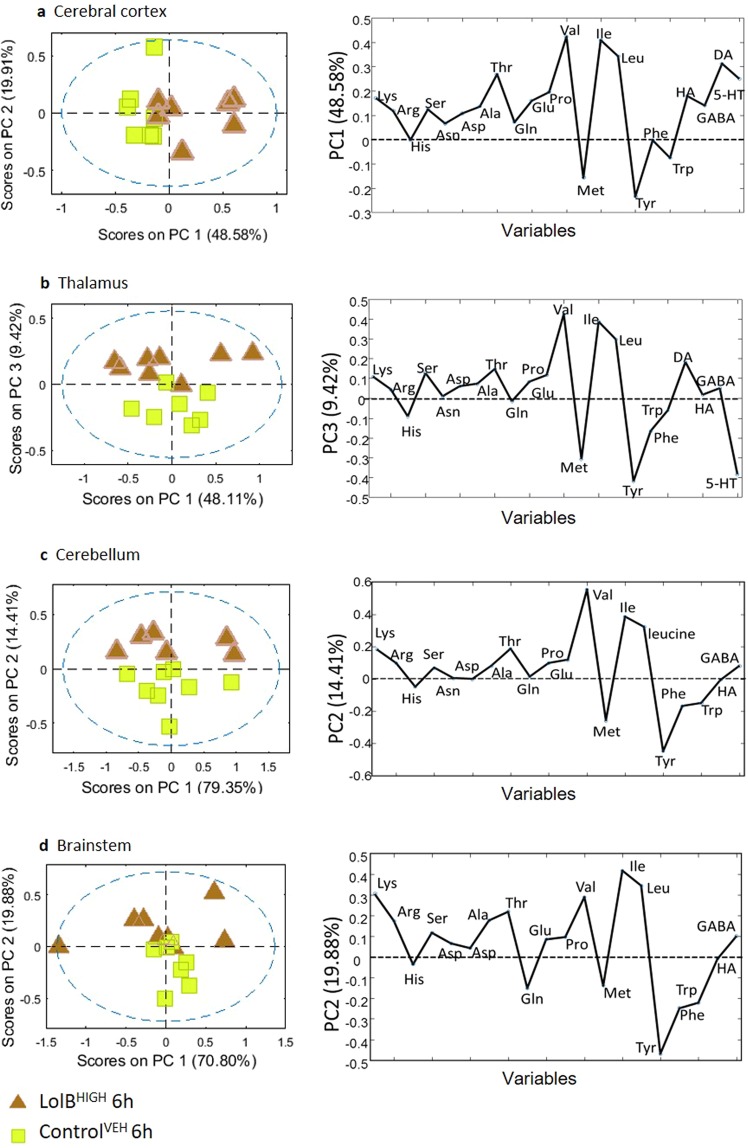
PCA and loadings plot of ESI + LCMS amino acid quantitation data acquired from aqueous extracts of individual brain tissue samples showing LolB^HIGH^ 6 h (*n = 7*) vs Control^VEH^ 6 h (*n = 7*). Conformation of the samples to the PCA model is shown as: [*Q* residuals, Hotelling *T*^2^]. These include **(a)** cerebral cortex [3.0%, 97.0%] **(b)** thalamus [9.7%, 90.3%] **(c)** cerebellum [6.3%, 93.8%] **(d)** brainstem [5.4%, 94.6%] tissues.

To determine the metabolic effects of lolitrem B, brain tissues were analysed using targeted LCMS. Metabolic variation with respect to amino acid and neurotransmitter expression was analysed using PCA score plots (Fig. 6) of the classes LolB^HIGH^ and Control^VEH^ at 6 h. None of the samples fell outside the 95% confidence limit (blue dashed line). Loadings plots were generated to identify differentially expressed metabolites contributing to the variation. Statistical significance of these data were determined by Student’s *t*-test.

The amino acid metabolites that exerted the greatest influence across all the brain regions include isoleucine, leucine and valine whose concentrations were increased compared to controls; tyrosine was decreased compared to controls. Also, with the exception of the brainstem, all other regions of the brain show a pronounced decrease in concentration of methionine.

The strong separation in the cerebral cortex could be attributed to the branched chain amino acids, valine (*p* = 0.0001), leucine (*p* = 0.0008) and isoleucine (*p* = 0.020) and the major catecholamine precursor, tyrosine (*p* = 0.039). Methionine (*p* = 0.022) also contributed to the separation illustrated in the PCA score plot. Compared to the Control^VEH^ the branched chain amino acid valine in the thalamus (valine, *p* = 0.038) and cerebellum (valine, *p* = 0.008) region was also significantly different. The discriminating metabolites in the thalamus region were tyrosine (*p* = 0.035) and methionine (*p* = 0.021). Branched chain amino acids (valine, *p* = 0.012; isoleucine, *p* = 0.024; leucine, *p* = 0.026) contributed to the separation in the brainstem region.

**Figure 7:**
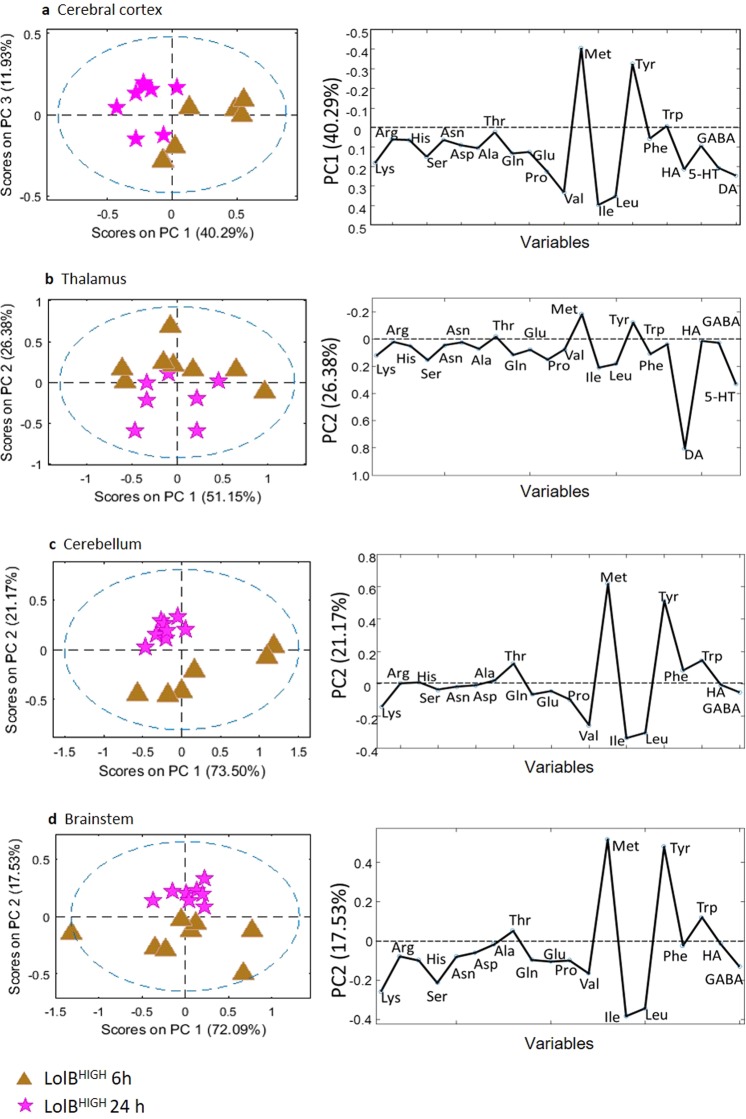
PCA and loadings plot of ESI + LCMS amino acid quantitation data acquired from aqueous extracts of individual brain tissue samples showing LolB^HIGH^ 6 h (*n = 7*) vs LolB^HIGH^ 24 h (*n = 7*). Conformation of the samples to the PCA model is shown as: [*Q* residuals, Hotelling *T*^2^]. These include (**a**) cerebral cortex [9.1%, 90.3%] (**b**) thalamus [4.3%, 95.7%] (**c**) cerebellum [9.0%, 90.9%] (**d**) brainstem [5.8%, 94.3%] tissues.

Temporal metabolic variation suggests metabolites return to a pre-treated state, except for threonine which maintained similar levels at LolB^HIGH^ 24 h compared to LolB^HIGH^ 6 h in all brain regions (Fig. 7). This is further supported by significantly increased levels of threonine across all brain regions for LolB^HIGH^ 24 h vs Control^VEH^ 24 h (cerebral cortex, *p* = 0.034; thalamus, *p* = 0.026; cerebellum, *p* = 0.049; brainstem *p* = 0.027). Together these data demonstrate the impact of lolitrem B intoxication on brain metabolism at peak tremor (6 h post injection), with lolitrem B exerting the greatest effect on the cerebral cortex.

**Figure 8:**
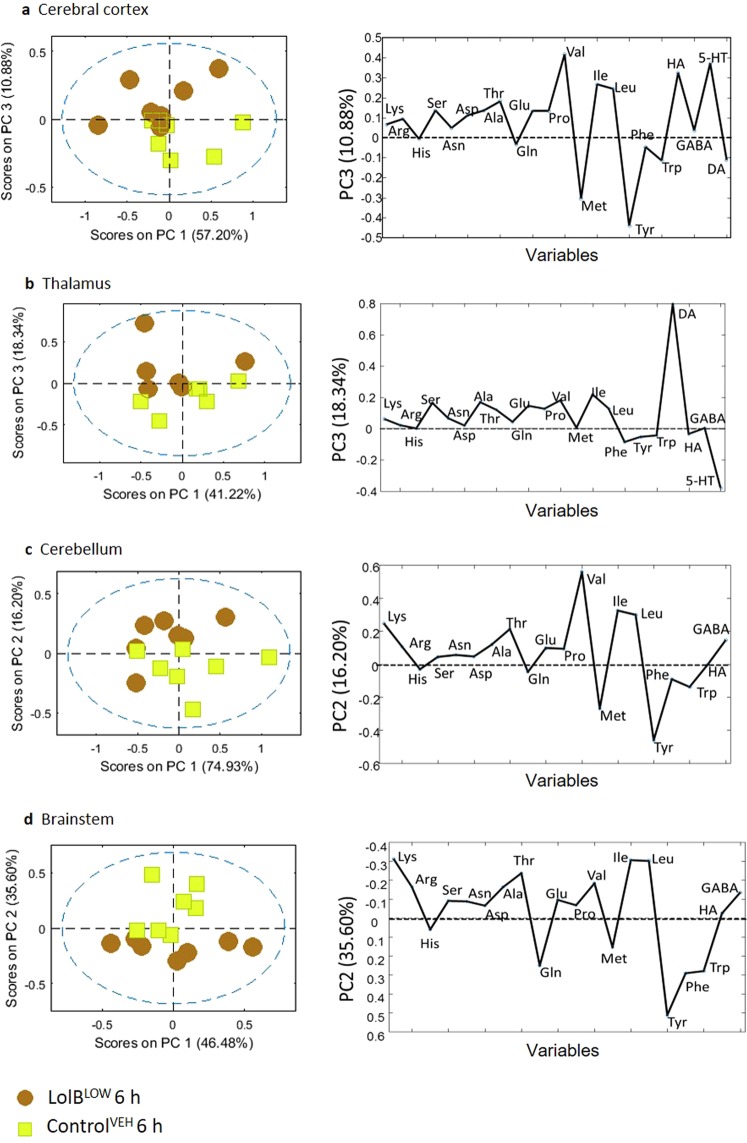
PCA and loadings plot of ESI + LCMS amino acid quantitation data acquired from aqueous extracts of individual brain tissue samples showing LolB^LOW^ 6 h (*n = 7*) vs Control^VEH^ 6 h (*n = 7*). Conformation of the samples to the PCA model is shown as: [*Q* residuals, Hotelling *T*^2^]. These include **(a)** cerebral cortex [9.9%, 90.2%] **(b)** thalamus [9.1%, 90.9%] **(c)** cerebellum [9.6%, 90.3%] **(d)** brainstem [10.4%, 89.6%] tissues.

To investigate effects of LolB^LOW^, PCA scores plots were generated (Fig. 8) of the classes LolB^LOW^ and Control^VEH^ 6 h. It can be observed that none of the samples fall outside the 95% confidence limit (blue dashed line). The low dose treated mice exhibit weaker variation compared to the high dose mice, however notable effects in the brain are clearly observed. Loadings plots (Fig. 8) show that differentially expressed metabolites in the low dose mice closely resembled the high dose treated mice. Isoleucine, leucine and valine concentrations increased compared to controls; tyrosine concentrations decreased compared to controls. With the exception of the thalamus, all the regions of the brain showed a pronounced decrease in methionine concentration.

**Figure 9:**
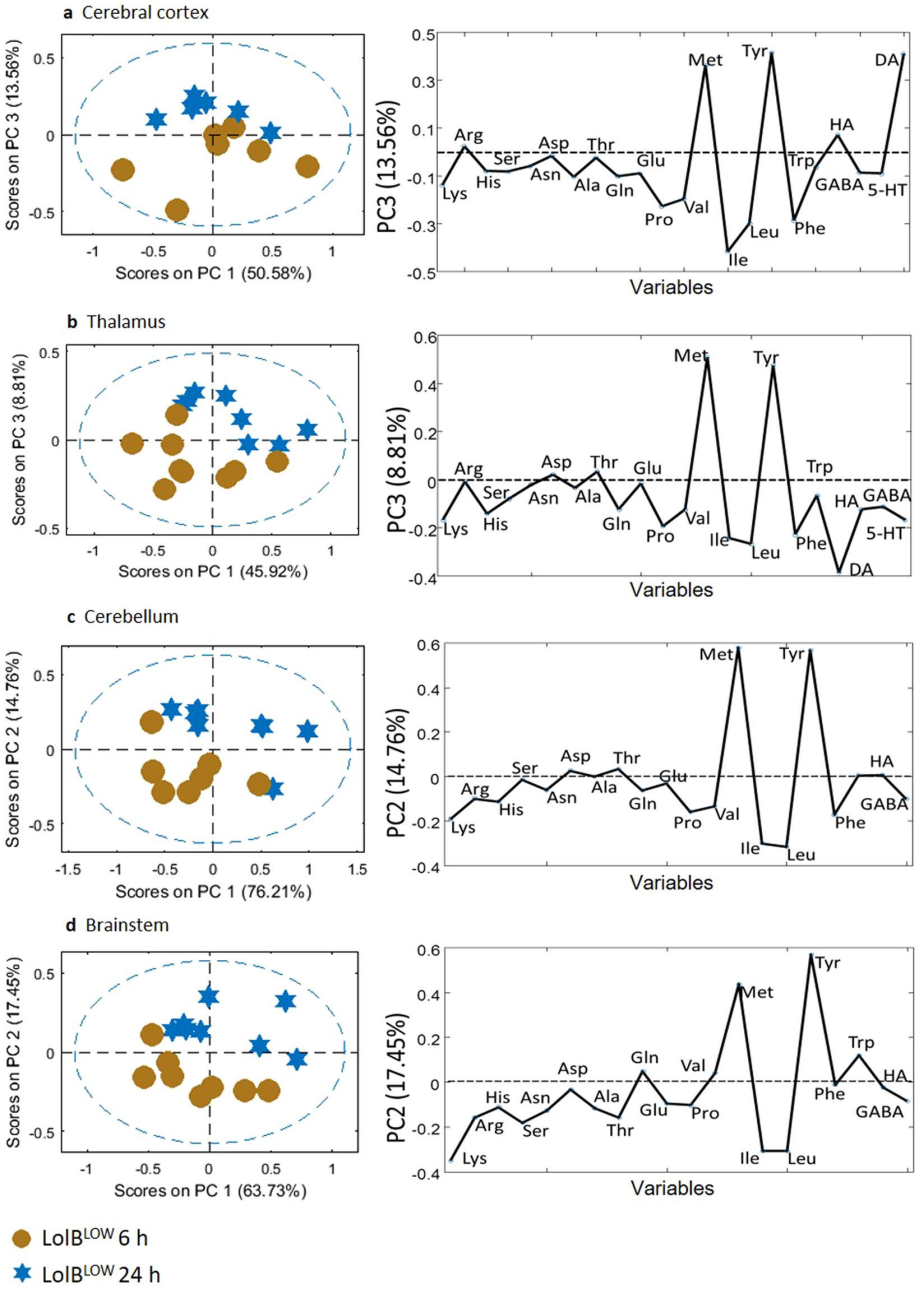
PCA and loadings plot of ESI + LCMS amino acid quantitation data acquired from aqueous extracts of individual brain tissue samples showing LolB^LOW^ 6 h (*n = 7*) vs LolB^LOW^ 24 h (*n = 7*). Conformation of the samples to the PCA model is shown as: [*Q* residuals, Hotelling *T*^2^]. These include **(a)** cerebral cortex [11.5%, 88.5%] **(b)** thalamus [15.4%, 84.6%] **(c)** cerebellum [9.0%, 90.9%] **(d)** brainstem [7.1%, 92.9%] tissues.

The significantly different metabolites in the cerebral cortex region are methionine (*p* = 0.038), tyrosine (*p = *0.038) and tryptophan (*p = *0.049). The thalamus region also showed significant changes in methionine (*p = *0.036) and tyrosine (*p = *0.018). Other metabolites in the thalamus region that showed pronounced variation are phenylalanine (*p* = 0.008), tryptophan (*p* = 0.028), histidine (*p* = 0.041), glutamine (*p* = 0.042) and GABA (*p* = 0.048). The brainstem showed significant changes in methionine (*p* = 0.039) and tyrosine (*p* = 0.030). Other metabolites also showed notable changes in the brainstem including threonine (*p* = 0.008), lysine (*p* = 0.028), tryptophan (*p* = 0.049) and histamine (*p* = 0.004). The significantly affected metabolite in the cerebellum was found to be tyrosine (*p* = 0.035). Temporal variation suggests metabolites return to a pre-treated state (Fig. 9).

The analysis suggests that a low dose of lolitrem B can still impact on brain metabolism, exhibiting similar metabolic variation with respect to amino acid and neurotransmitter expression compared to high dose treated mice in the time points studied. Thus, these data provide further confirmation that lolitrem B intoxication triggers metabolic variation in a dose-dependent manner.

PCA loadings plots indicated that branched chain amino acids (valine, isoleucine and leucine) and tyrosine and methionine strongly influence separation between vehicle control and treatment groups. Thus ROC (receiver-operating characteristic) curves were used to evaluate the specificity and sensitivity; sensitivity and 1-specificity corresponded to the true positive rate and false positive rate, respectively^34^ in all the brain regions exposed to LolB^HIGH^ and compared to Control^VEH^ from all brain regions. AUCs have been applied as useful indices for discriminating the diagnostic values of features. If the AUC is greater than 0.7, the features are regarded as useful biomarkers^35–38^. The AUC values which are greater than 0.7 of AUC substances values are considered to be more sensitive and specific. The analysis resulted in five biomarkers that showed AUCs greater than 0.7 and ranged from 0.70–0.78, as shown in Table 2. (Extended Data is found in Supplementary Table S2 to S5 online).

**Table 2:** ROC curve Analysis of Biomarkers for LolB^HIGH^ 6 h exposed mice (all brain regions) (Supporting information is in Supplementary Data Tables S1–S4).

Metabolite	All Brain Regions: Control^VEH^ vs LolB^HIGH^					
	AUCs	Sensitivity (%)	Specificity (%)	95% Confidence Interval	T-tests	KM Cluster
Leucine	0.78	70%	70%	0.65–0.90	3.7E-4	5.0
Methionine	0.74	80%	70%	0.62–0.87	4.1E-3	1.0
Isoleucine	0.74	60%	80%	0.61–0.86	9.0E-4	5.0
Tyrosine	0.74	80%	70%	0.61–0.86	1.3E-3	1.0
Valine	0.70	60%	80%	0.57–0.83	1.8E-3	2.0

**Figure 10:**
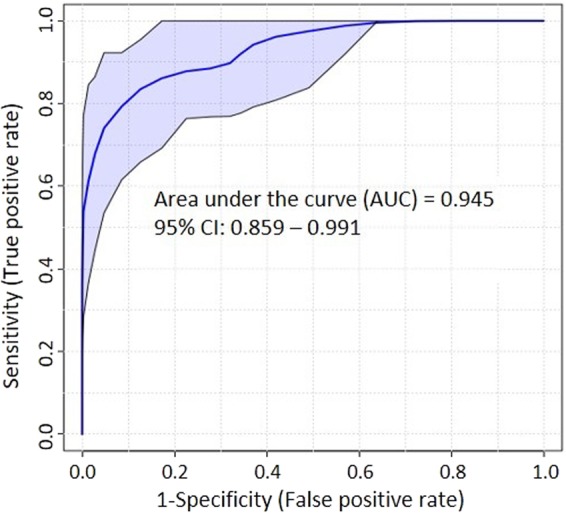
ROC curve of top 4 features (Table 2) showing AUC = 0.95 and a sensitivity and specificity at a 95% confidence interval of 0.86–0.99.

To evaluate the predictive performance of the combination of biomarkers, Linear SVM was applied. Based on KM Clustering 4 out of the 5 top biomarkers were selected to build a classification model. The ROC curve generated from the model resulted in an AUC of 0.95 and 0.859–0.991 at 95% confidence interval (Fig. 10). The cross-validation accuracy of the model showed good prediction performance of 0.85 based on 100 cross validations. The predictive performance of the model was found to be significant (P < 0.000448). The accuracy of the results demonstrated that the SVM model can be used as a diagnostic tool to determine lolitrem B exposure in brain tissue of animals. The reliability of the model was tested on LolB^LOW^ brain regions. The SVM model predicted the brainstem region with 75% accuracy (6 out of 8 replicates of LolB^LOW^ 6 h); cerebral cortex and thalamus with 71.4% accuracy (5 out of 7 replicates of LolB^LOW^ 6 h) and cerebellum with 42.8% accuracy (3 out of 7 replicates of LolB^LOW^ 6 h). These results indicate that the SVM model based on the combinatorial biomarkers could effectively predict mild to severe cases of lolitrem B intoxication across most brain regions.

## Discussion

This study investigated movement and coordination using sensitive screening platforms that enable accurate and effective measures of tremorgenicity and motor coordination deficits in *in-vivo* models. The indole diterpene class of compounds is known to display biological activities based on very specific conformational, structural and stereo-specific features^25,33^. The indole diterpenes studied exhibited biological activities consistent with previous reports^5,17,32,39^. Terpendole B had not been previously tested and, like terpendole E, showed no tremorgenic activity and did not affect coordination or movement in exposed mice. Our results show that rotarod, motor performance and tremor tests provide an effective and sensitive way of measuring indole diterpenoid toxicity in mice in a dose-dependent manner.

To elucidate the mode and site of action of the indole diterpenoid toxins examined in this study, tremor and rotarod performance was coupled to metabolomics profiling using ESI + LCMS quantitation analysis to determine the effect of lolitrem B exposure on brain metabolism in C57Bl/6J mice at two time points, 6 h and 24 h, post treatment. Our results show that lolitrem B caused a profound disruption in metabolic profile across all regions of the brain, but particularly the cerebral cortex at 6 h and were coincident with high levels of lolitrem B in this brain region.

Recently, the clinical signs presenting in ruminants exposed to lolitrem B toxin have been defined as those of a central rather than cerebellar tremor^7^. Our findings also support a hypothesis that the movement deficits observed in these animals are upstream of the cerebellum, with the cerebellar lesions being secondary via super-excitation of efferent fibres entering the cerebellum from the cranial nerve nuclei or via subcortical movement centres. The highly lipophilic nature of lolitrem B allows for strong association and accumulation of the toxin in fatty tissues^40–42^. Strong bioaccumulation, and slow removal of lolitrem B from the brain explains the prolonged tremors observed in animals subsequent to ingestion of toxic pastures. Dysregulation of neurotransmitters and amino acids was observed at 6 h in high and low dose lolitrem B treated mice correlating with this bioaccumulation profile.

Effects of toxicity were also observed in a dose-dependent manner with low doses of lolitrem B exerting a lower tremorgenic effect than high doses in mice in this study. Conversely, concentrations of lolitrem B in all brain regions examined at both doses, and both timepoints analysed did not show significant differences, indicating that both low and high levels of toxic exposure cause significant bioaccumulation of toxin in the brain over a short period of time. This bioaccumulation was not, however, associated with significant changes in metabolic profile except in the cerebral cortices.

Metabolomic profiling identified that the metabolites exerting the majority of the effect at 6 h were isoleucine, leucine, valine, tyrosine and methionine. Isoleucine, leucine, and valine were increased compared to controls, with tyrosine and methionine decreased compared to controls. Decreased levels of tyrosine, the precursor of dopamine, may influence catecholamine synthesis. Low levels of dopamine in Parkinson’s disease and similar central neurodegenerative disorders are associated with symptoms of hypokinesia, tremor, rigidity, and abnormal posture^43^ similar to those observed in lolitrem B intoxicated animals. The branched-chain amino acids leucine, isoleucine and valine have important biochemical functions in the brain. Particularly, the synthesis of serotonin, dopamine and norepinephrine, which are derived from the aromatic amino acids tryptophan, tyrosine and phenylalanine respectively^44–50^. These have wide-ranging effects across multiple neurological systems, including those associated with emotion and behaviour, alterations that are observed in lolitrem B intoxicated animals^7^.

Branched-chain amino acids compete for transport across the blood-brain barrier with aromatic amino acids. It has been reported that a rise in plasma branched-chain amino acid concentrations are known to have a direct effect on the synthesis and the release of biogenic amines derived from these aromatic amino acids, notably serotonin (from tryptophan) and catecholamines, dopamine and norepinephrine (from phenylalanine and tyrosine), in the brain^44–50^. Increased levels of branched-chain amino acids found in metabolic diseases such as maple syrup urine disease results in serious neurological disorders such as ataxia and lethargy^51,52^.

Metabolism of complex compounds and amino acids occurs primarily in the liver. However, many branched-chain amino acids are catabolised in non-hepatic tissues such as cardiac muscle and kidney^53,54^. The kidney, heavily intoxicated with lolitrem B at 6 h, could have had impaired capacity to catabolise branched-chain amino acids which may then accumulate. This may be the cause of the increase in these amino acids in the brain as observed in this study and be directly affecting catecholamine synthesis pathways in the brain.

**Figure 11:**
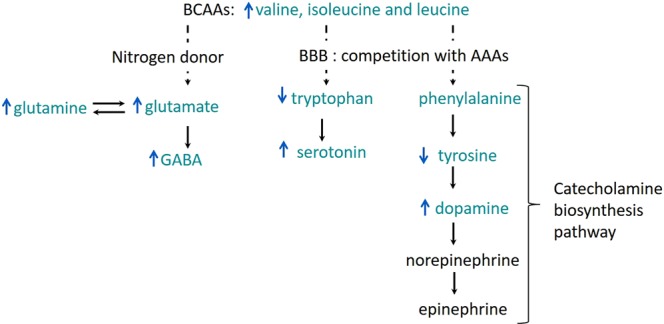
A schematic of the metabolic response to lolitrem B intoxication in the brain. Shown here are commonly affected pathways. The blue arrows represent the direction (increase/decrease in abundance) of metabolites in the cerebral cortex of LolB^HIGH^ 6 h. BBB refers to blood-brain barrier and AAA refers to aromatic amino acids. BCAA refers to branched chain amino acids.

A summary of the major metabolic pathways perturbed in the cerebral cortex, identified in our analysis, resulting from lolitrem B exposure is shown in Fig. 11. The relationship between branched-chain amino acids and aromatic amino acids is well known^44–50^. In this study we observed a strong reduction in levels of tyrosine and mild decreases in tryptophan and phenylalanine with variable expression of serotonin and dopamine with region and time-dependent differences. Cellular differences are, however, not measurable by this analysis and only changes in the overall metabolomic pool can be detected. Since neurotransmitters are present in such low abundances, small changes at localised sites are not reliably detected^55,56^. Further experiments would be required to understand the effect of lolitrem B at a cellular level.

Decreased levels of methionine across all brain regions and treatments were observed in this study^57^. Methionine has been reported to modify dopamine levels and alleviate symptoms of Parkinson’s disease^58^. However, excess levels of *S*-adenosylmethionine (SAM), a precursor of methionine, has also been reported to deplete dopamine levels, inducing symptoms characteristic of Parkinson’s disease^43^. Therefore alteration in this metabolic pathway could be underlying the tremor phenotype observed in the mice in this study. In a pathological state, such as infection and cancer^59^, amino acids as substrates are in high demand for energy production^59^. Mice in this study exhibited an overall increase in amino acids, particularly in the cerebral cortex. The strong metabolic variation induced by lolitrem B is either implicated in the catecholamine pathway or neuromodulatory reaction, suggesting increased excitatory activity in the brain.

The temporal variation in the metabolome observed in this study shows that whilst peak tremor (6 h) triggers a clear perturbation in multiple pathways, the majority of those key metabolites show a clear return to a pre-treated state at 24 h. This correlates with the tremor profile in these mice where tremor was visibly reduced at 24 h compared to 6 h post exposure, suggesting a moderation of neurotransmitter production or recycling, despite no observable difference in toxin concentrations. Study of the binding kinetics of lolitrem B and its pathway intermediates to BK channel receptors would further elucidate the mode of action of this toxin and its potential uses for pharmacological purposes.

In conclusion, this study is the first functional metabolomics analysis of any naturally occurring toxin in a rodent model. Our data identified lolitrem B synthetic pathway members that had both tremorgenic and non-tremorgenic activity. Key differences were identified in concentration of the toxin in different parts of the brain, with highest concentrations observed in the cerebral cortex and levels below the limit of detection in the cerebellum. Metabolomics profiling identified key differences in neurotransmitter and amino acid profiles between different brain regions, and over time, with greatest differences observed at 6 h post exposure, the point of greatest tremor intensity. Dysregulation of catecholaminergic neurotransmitter pathways appear to be delivering the majority of effect in the forebrain with both up and down dysregulation of key biosynthetic precursors identified. Future studies should be undertaken to further understand the bioaccumulation of lolitrem B toxin in the brain and its receptor binding kinetics in order to determine potential pharmacological uses for this potent family of neuroactive toxins.

## Materials and Methods

### Toxins

Lolitrem B and terpendole B were isolated and purified as previously described^60^ and were shown to be at least 98% pure by NMR spectroscopy. Terpendole C (98% pure) was purchased from Jomar Life Research (sc-391042) and terpendole E (98% pure) sourced from Sigma Aldrich (SML1127). All toxins were administered by intraperitoneal (i.p.) injection in the vehicle carrier 9:1 (v/v) dimethyl sulfoxide (DMSO: H_2_O)^33^.

Lolitrem B was administered at 0.5 (LolB^LOW^) and 2.0 mg/kg (LolB^HIGH^). These doses correspond to hypothesized subclinical (low) doses and known disease-inducing (high) concentrations subsequent to ingestion in pasture-fed herbivores^61^. Terpendole C was administered at 2.0 (TerpC^LOW^) and 4.0 mg/kg (TerpC^HIGH^) and terpendole E at 8 (TerpE^LOW^) and 16 mg/kg (TerpE^HIGH^). Terpendole B was only available in very small quantities and a single dose was therefore used: 0.5 mg/kg (TerpB). Two negative control groups were used for comparison of tremor and behaviour: (1) an untreated control (Control), and (2) a vehicle control (Control^VEH^) which had been treated with the intraperitoneal (i.p.) injection vehicle 9:1 (v/v) DMSO: H_2_O only.

### Behavioural tests

All mouse studies were approved by the La Trobe University Animal Ethics Committee (Protocol number 15–87) and were conducted in accordance with the Australian Code of Practice for the Care and Use of Animals for Scientific Purposes set out by the National Health and Medical Research Council of Australia. The mice were housed in groups of two to four during the experimental period in individually-ventilated cages (Tecniplast, Buguggiate, Italy) with standard pellet food and water available *ad libitum*. Ambient temperature of housing and testing rooms was 21 ± 2 °C and mice were housed under a 12 h light–dark cycle (lights on at 7 am). A total of 184 male 8–9 week old C57Bl/6J mice were sourced from a breeding colony at the Walter and Eliza Hall Institute of Medical Research, Melbourne, Victoria. Mice were allowed to acclimatise to the facility conditions for a period of 1 week prior to behavioural testing. All mice underwent a series of behavioural tests to examine effects of intoxication on tremor and movement.

To capture peak neurotoxic response and measure return to baseline for both tremor analysis and coordination^5^, tremor testing and rotarod tests for lolitrem B treated mice were conducted in 40 min intervals for 12 h, then again at 24 h post treatment. For terpendole C, measurements were conducted at 12 min intervals for 2 h post treatment. For terpendole B and E, tremor and rotarod activity were undertaken at 40 min intervals for 8 h post treatment before final testing and euthanasia at 30 h.

### Tremor analysis

To measure frequency and amplitude of tremor associated with intoxication, mice were placed individually into a closed container mounted on a piezo-electric pulse transducer sensor (ADI Technologies, USA) to convert force applied to the surface of the transducer into an analog signal^62^. Data from the sensor were converted through an AC amplifier (model DP-311; Grass Instruments, West Warwick, RI) with a bandpass of *1–50 *Hz applied and a sampling rate of 100 samples/s. Motion power output was recorded digitally for 3 min and analysed for change of frequency and power using LabChart™ 7.0 (ADInstruments, Castle Hill, Australia).

**Figure 12:**
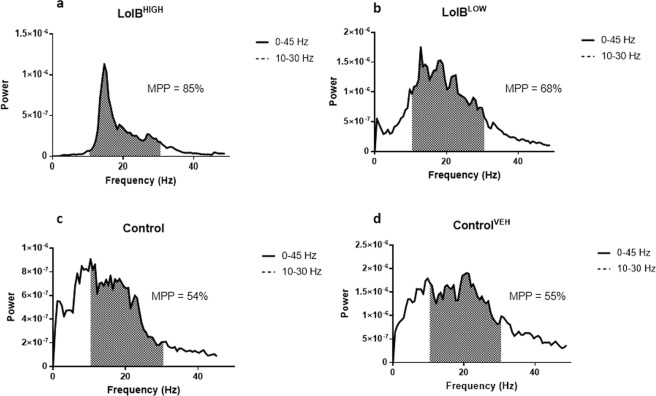
The power output spectra of **(a)** LolB^HIGH^ and **(b)** LolB^LOW^ mice exhibiting tremors compared to **(c)** Control and **(d)** Control^VEH^ mice. The tremor was defined by the AUC of tremor frequency bandwidth divided by AUC of overall motion power [100* (10–30 Hz power)/(0–45 Hz power)]. This ratio is expressed as motion power percentage (MPP) and in untreated mice; the MPP is approximately 40 to 55%, corresponding not to any visible tremor but to normal motion within these bandwidths.

Raw data (Time vs Frequency) for each 3 min epoch were converted to a power output spectrum (Frequency (Hz) vs. Power (V^2^)) between 0 and 45 Hz by Fast-Fourier-Transformation (FFT). Control mice showed peak activity defined by a 0 and 10 Hz bandwidth, representing normal movement. All tremorgenic mycotoxins tested induced a prominent peak in the motion power spectrum between 10 and 30 Hz, with reduction in power output at other frequencies (Fig. 12). Tremor, intensity was therefore able to be defined by the area under the curve (AUC) of tremor frequency bandwidth divided by AUC of overall motion power [100* (10–30 Hz power)/(0–45 Hz power)]. This ratio was expressed as motion power percentage (MPP). In untreated mice, MPP corresponding to normal motion was observed to be 40–55% and values above 60% are indicative of tremor.

### Coordination analysis

To measure motor coordination and balance, mice undertook sequential testing on an accelerating rotarod (Rotamex, Columbus Instruments, Columbus, OH 43204, USA). Mice were first trained to use the apparatus over 8–10 trials, with 20 min between each trial, or until 3 stable baseline performances had been achieved. For each test, acceleration was applied from 4 to 40 rpm over 120 s then held constant for a further 60 s and latency to falling recorded. Mice that rotated passively were deemed to have fallen.

### Metabolomic profiling

Brain tissues of mice exposed to low (0.5 mg/kg) or high (2.0 mg/kg) doses of lolitrem B were collected at either 6 h or 24 h post-treatment and compared to untreated controls or vehicle injected controls (n = 8 at each time point) by metabolomic analysis. To confirm the intoxication phenotype was consistent to previous analyses, all mice were subjected to tremor testing 5 min prior to euthanasia and tremor data compared to data collected from the behavioural analysis described above. Mice were euthanized by cervical dislocation and tissues were collected, including liver, kidney, and the brain (separated into cerebral cortex, thalamus, cerebellum and brainstem). Samples were weighed and snap-frozen in liquid N_2_ before storage at −80 °C for metabolomics analysis.

Frozen samples of kidney, liver, thalamus and cerebral cortex were transferred into 4 mL polycarbonate tubes with 3/8″ stainless steel grinding balls and kept frozen in liquid nitrogen. Sample tubes were placed into pre-frozen 24 well cryo-blocks on the Geno/Grinder 2010 (SPEX Sample Prep, Metuchen, NJ, USA) and the tissues were homogenised at 1,750 rpm for 1 min. The fine powder was stored at −80 °C until ready to be weighed. Kidney and liver samples (50–52 mg) as well as cerebral cortex and thalamus (20–22 mg) samples were each weighed in 2 mL Eppendorf tubes. Liver, kidney, cerebral cortex and thalamus samples of known weight were extracted using a modified Bligh-Dyer extraction method^63^. Briefly, methanol:H_2_O (1.6:0.6, v/v) was added to tissue powder and vortex-mixed prior to addition of dichloromethane (DCM) (Burdick & Jackson, HPLC grade). Samples were then sonicated on ice before addition of 1:1 v/v DCM:H_2_O. Samples were centrifuged for 10 min at 13,000 rpm and the upper aqueous layer transferred into HPLC vials ready for LCMS analysis of polar metabolites. An aliquot (10 μl) of each sample was combined to generate a pooled biological quality control (PBQC) sample, which was used to monitor analytical reproducibility. Two PBQC samples were prepared for each tissue type (cerebral cortex and thalamus). The solvent layer was then transferred to a separate tube and evaporated under a stream of N_2_. Solvent extracts were also analysed to determine lolitrem B concentration by LCMS.

The sample sizes of the cerebellum and brainstem were smaller (30–70 mg) than other brain regions collected so a modified method was used. Cerebellum and brainstem were placed directly in pre-weighed Lysing matrix D tubes (MP Biomedicals, product number 116913050) prefilled with 1.4 mm ceramic beads. For homogenization, 1 mL of cold 4:1 v/v methanol: H_2_O was added to each sample and placed in a Precellys 24-tissue homogeniser (PEQLAB Biotechnology GmbH, Germany) fitted to a Cryolys N_2_ Cooling System. When the temperature reached −20 °C, samples were homogenized at a speed of 5,500 rpm over 3 consecutive 20 s cycles with 30 s intervals. Cerebellum and brainstem were subsequently extracted with 4:1 v/v methanol: H_2_O (40 µL/mg sample) and the aqueous phase transferred into HPLC vials with inserts for LCMS analysis. Quality controls were prepared as before. In addition, an aliquot of the remaining extract was evaporated under N_2_ before freeze drying for NMR analysis.

LCMS data were analysed in Thermo Xcalibur Qual Browser v.2.3.26 (Thermo Fisher Scientific™). Assessment of peak retention time and ion extraction window (m/z) confirmed the presence of the targeted polar compounds within the standard mixes.

### Metabolite quantitation

All extracts were analysed on a Vanquish Ultra-High Performance Liquid Chromatography (UHPLC) system (Thermo Fisher Scientific, Bremen) with a binary pump, autosampler and temperature-controlled column compartment coupled with a QExactive (QE) Plus mass spectrometer (Thermo Fisher, Waltham, MA, USA; Thermo, Bremen, Germany) detector. The Thermo Fisher QExactive Plus mass spectrometer was set at FT switching positive and negative mode over a mass range of 70–1,200 amu with resolution set at 35,000. Nitrogen was used as the sheath, auxillary and sweep gas at a flow rate of 28, 15 and 4 L/min, respectively and spray voltage was set at 3,600 V (positive) and 3,300 V (negative). Samples were randomized, and blanks were run every 5 samples. A PBQC was run every 10 samples. Prior to data acquisition, the system was calibrated with Pierce® LTQ Velos ESI Positive and Negative Ion Calibration Solution (Thermo Fisher Scientific™). Mass spectrometry data was acquired using Thermo Xcalibur V. 2.1 (Thermo Fisher Scientific Inc., USA). Quantitative analysis was conducted using LCQUAN™ Quantitative Software (Thermo Fisher Scientific™).

Polar metabolites were eluted from the column (Phenomenex 250 × 4.6 mm 4 µm Synergi HPLC column) using a gradient mobile phase, A (0.1% formic acid: H_2_O), and B (0.1% formic acid: acetonitrile) at 0.5 mL/min with 98% A to 0% A over 11 min with a linear gradient. All amino acids and neurotransmitter standards were purchased from Sigma-Aldrich. For quantitative analysis, standard curves were prepared in H_2_O for alanine (Ala), asparagine (Asn), aspartic acid (Asp), arginine (Arg), glutamic acid (Glu), glutamine (Gln), histidine (His), isoleucine (Ile), leucine (Leu), lysine (Lys), methionine (Met), phenylalanine (Phe), proline (Pro), serine (Ser), threonine (Thr), tryptophan (Trp), tyrosine (Tyr) and valine (Val) and 50% acetonitrile/water for 10 biogenic amines: dopamine (DA), epinephrine, GABA, histamine (HA), N-methylphenethylamine, norepinephrine, octopamine, phenethylamine, serotonin (5-HT) and tryptamine in a range of concentrations from 0.0001 to 30 µg/mL.

To determine concentrations of lolitrem B, reconstituted tissue extracts were eluted from the column (Agilent 150 × 2.1 mm, 3.5 µm Zorbax Eclipse XDB-C8 HPLC column) using a gradient mobile phase, A (0.1% formic acid in H_2_O) and B (0.1% formic acid in 55% isopropanol (IPA) in acetonitrile) at 0.5 mL/min with 98% to 0% A over 11 min. Two standard curves were prepared for lolitrem B, a high concentration range of 6 to 2400 ng/mL and low concentration range from 0.5 to 60 ng/mL. Assessment of peak retention time and ion extraction window (m/z) on Thermo Xcalibur Qual Browser v.2.3.26 (Thermo Fisher Scientific™) confirmed the presence of lolitrem B. Spike recovery analysis was performed for 300 and 600 ng/mL standards of lolitrem B for each tissue type in duplicate. Limit of detection (LoD) of lolitrem B by this method was 0.8 ng/mL.

### Statistical analysis and bioaccumulation modelling

For the behavioural analysis, statistical significance was assessed by using GraphPad Prism 7.04 (GraphPad Software, Inc., La Jolla, CA). Data were analyzed by two-way ANOVA followed by uncorrected Fisher’s LSD test for multigroup comparisons against vehicle control. Data are expressed as the mean ± SEM. The threshold of *P* < 0.05 was designated as statistically significant for all tests.

For metabolomics analysis, data were normalised by log transformation (log 10) and mean centering pre-processing. *P* values were calculated using unpaired Student’s *t*-test data using the MetaboAnalyst 3.0^64^. The threshold of *p* < 0.05 was designated as statistically significant for all tests. A multivariate statistical approach was also employed to assess differentially expressed LCMS targeted metabolites on the normalized data. An unsupervised principal component analysis (PCA) was carried out on the quantitation data in MatLab using PLStoolbox_731. The Hotelling *T*^2^ statistic and the *Q* residuals were used to indicate how well each sample conformed to the PCA model as well as detect residual outliers in the datasets. The optimum values of the *Q* residuals and the Hotelling *T*^2^ value are 0% and 100%, respectively^65^. The *Q* residual is a measure of the difference, or residual, between a sample and its projection into the *k* principal components used to build the model^66^. The Hotelling *T*^2^ statistic is a measure of the variation in each sample within the PCA model, or otherwise defined as a measure of the distance from the multivariate mean to the projection of the sample onto the *k* principal components^65,67^.

To discriminate effects between control and treatment groups a Univariate Receiver Operating Characteristic (ROC) Curve analysis was applied using MetaboAnalyst 3.0. A prediction model was developed, and data were log2 transformed/normalised with no scaling applied. ROC curve model evaluation was selected, and the top four features were identified for each treatment (Table 2). The ROC area under the curve (AUC) and *t* tests (*p* < 0.05) for metabolite ratios for each tissue were ranked based on AUC values. All specific biomarker candidates were subjected to linear SVM (support vector machine) to establish a statistical prediction model.

## Supplementary information

Supplementary information

## Data Availability

The datasets generated during the current study are available from the corresponding author on reasonable request.
