# Supplementary material for: Tremorgenic effects and functional metabolomics analysis of lolitrem B and its biosynthetic intermediates
Source: Sci Rep. 2019 Jun 27;9:9364. doi: 10.1038/s41598-019-45170-7 (PMC6597573; doi:10.1038/s41598-019-45170-7)
Supplement: Supplementary file 1 — Supplementary information [file 41598_2019_45170_MOESM1_ESM.docx]

Title: Tremorgenic effects and functional metabolomics analysis of lolitrem B and its biosynthetic intermediates

Abbreviated title: Functional metabolomics of lolitrem B toxin in mouse brain

Priyanka Reddy^1,2,^, Simone Rochfort*^1,2^, Elizabeth Read^1,2^, Myrna Deseo^1^, Emily Jaehne^3^, Maarten Van Den Buuse^3^, Kathryn Guthridge^1^, Martin Combs^4,5^, German Spangenberg^1,2^, and Jane Quinn^4,5^

**Table S1** Recovery rates are also shown of lolitrem B spiked at 2 concentration levels, 54 ng/mL and 109 ng/mL in various tissue samples.

| **Tissue** | **Low concentration**  **spike (54 ng/mL)**  **% recovery ±SD** | | | **High concentration**  **spike (109 ng/mL)**  **% recovery ±SD** | | |
| --- | --- | --- | --- | --- | --- | --- |
| **cerebral cortex** | 96.2 | ± | 6.2 | 93.4 | ± | 8.2 |
| **thalamus** | 107.0 | ± | 0.5 | 91.7 | ± | 8.8 |
| **cerebellum** | 88.1 | ± | 0.7 | 85.5 | ± | 4.5 |
| **brainstem** | 87.5 | ± | 0.6 | 83.1 | ± | 4.7 |
| **liver** | 95.7 | ± | 8.8 | 95.4 | ± | 7.1 |
| **kidney** | 91.2 | ± | 4.4 | 100.5 | ± | 1.4 |

**Table S2** ROC curve Analysis of Biomarkers for LolB^HIGH^ exposed mice (cerebral cortex).

| Metabolite | Cerebral cortex: LolB^HIGH^ vs Control^VEH^ 6 h | | |
| --- | --- | --- | --- |
|  | AUCs | T-tests | Log2FC |
| Valine | 1.0 | 0.0001 | 0.96 |
| Leucine | 0.92 | 0.0008 | 0.53 |
| Methionine | 0.90 | 0.0224 | -0.60 |
| Isoleucine | 0.86 | 0.0199 | 0.64 |
| Tyrosine | 0.83 | 0.0394 | -0.72 |
| Threonine | 0.79 | 0.0539 | 0.35 |
| Proline | 0.79 | 0.1178 | 0.25 |
| Tryptophan | 0.76 | 0.1582 | -0.31 |
| Serotonin | 0.75 | 0.1439 | 0.25 |
| Lysine | 0.71 | 0.2809 | 0.16 |

**Table S3** ROC curve Analysis of Biomarkers for LolB^HIGH^ exposed mice (thalamus).

| Metabolite | Thalamus: LolB^HIGH^ vs Control^VEH^ 6 h | | |
| --- | --- | --- | --- |
|  | AUCs | T-tests | Log2FC |
| Methionine | 0.84 | 0.0211 | -0.58 |
| Valine | 0.82 | 0.0378 | 0.46 |
| Tyrosine | 0.78 | 0.0351 | -0.76 |
| Phenylalanine | 0.73 | 0.1155 | -0.43 |
| Histidine | 0.71 | 0.1497 | -0.19 |
| Asparagine | 0.71 | 0.1863 | -0.13 |
| Tryptophan | 0.71 | 0.1335 | -0.35 |

**Table S4** ROC curve Analysis of Biomarkers for LolB^HIGH^ exposed mice (cerebellum)

| Metabolite | Cerebellum: LolB^HIGH^ vs Control^VEH^ 6 h | | |
| --- | --- | --- | --- |
|  | AUCs | T-tests | Log2FC |
| Valine | 0.90 | 0.0196 | 0.69 |
| Leucine | 0.83 | 0.0717 | 0.45 |
| Isoleucine | 0.79 | 0.1308 | 0.50 |
| Threonine | 0.76 | 0.1096 | 0.26 |
| Methionine | 0.71 | 0.1752 | -0.56 |

**Table S5** ROC curve Analysis of Biomarkers for LolB^HIGH^ exposed mice (brainstem)

| Metabolite | Brainstem: LolB^HIGH^ vs Control^VEH^ 6 h | | |
| --- | --- | --- | --- |
|  | AUCs | T-tests | Log2FC |
| Valine | 0.88 | 0.0115 | 0.47 |
| Isoleucine | 0.86 | 0.0236 | 0.49 |
| Leucine | 0.84 | 0.0259 | 0.40 |
| Serine | 0.77 | 0.0496 | 0.22 |
| Threonine | 0.77 | 0.0786 | 0.23 |
| Methionine | 0.71 | 0.2996 | -0.21 |
| Tyrosine | 0.71 | 0.0946 | -0.49 |
